# A Combined Thin Film/Thick Film Approach to Realize an Aluminum-Based Strain Gauge Sensor for Integration in Aluminum Castings

**DOI:** 10.3390/s20123579

**Published:** 2020-06-24

**Authors:** Rico Tiedemann, Dennis Lepke, Martin Fischer, Christoph Pille, Matthias Busse, Walter Lang

**Affiliations:** 1 Microsystems Center Bremen, Institute for Microsensors, Actuators and Systems (IMSAS), University of Bremen, 28359 Bremen, Germany; dlepke@uni-bremen.de (D.L.); wlang@imsas.uni-bremen.de (W.L.); 2 Research Group Near-net-shaping Production Technologies, Faculty of Production Engineering, University of Bremen, 28359 Bremen, Germany; martin.fischer@ifam.fraunhofer.de (M.F.); busse@ifam.fraunhofer.de (M.B.); 3 Fraunhofer Institute for Manufacturing Technology and Advanced Materials (IFAM), Wiener Straße 12, 28359 Bremen, Germany; christoph.pille@ifam.fraunhofer.de

**Keywords:** aluminum-based sensor, strain gauge, sensor integration, thin-film/thick-film combination, metal casting, gravity die casting, smart part, structural health monitoring

## Abstract

There is currently a large demand for aluminum components to measure the mechanical and thermal loads to which they are subjected. With structural health monitoring, components in planes, vehicles, or bridges can monitor critical loads and potentially prevent an impending fatigue failure. Externally attached sensors need a structural model to obtain knowledge of the mechanical load at the point of interest, whereas embedded sensors can be used for direct measurement at the point of interest. To produce an embedded sensor, which is automatically encapsulated against environmental influence, the sensor must be able to withstand the boundary conditions of the host component’s manufacturing process. This embedding process is particularly demanding in the case of casting. Previous work showed that silicon-based sensors have a high failure rate when embedded in cast aluminum parts and that using aluminum as a substrate is preferable under these circumstances. In the present paper, we present the fabrication process for the combination of a thick-film insulation and a thin-film strain gauge sensor, on such an aluminum substrate. The sensor is capable of withstanding high temperatures of at least 600 °C for over 20 min and a subsequent embedding in a gravity die casting process.

## 1. Introduction

Structural components made of aluminum are not currently equipped to monitor the mechanical loads to which they are subjected and an overload can only be detected by fatigue or an inspection. State-of-the-art aluminum components used for structural health monitoring include externally mounted strain gauges. Mounted sensors are often exposed to elements such as harsh weather conditions, saltwater, or physical damage, and are thus encapsulated with special care. Structural health monitoring has so far mainly been a topic within aerospace contexts [1]. Smart aluminum components, which are able to monitor their thermal and mechanical state with embedded sensors, will add value to critical applications where the detection and measurement of mechanical overload is necessary.

Embedding sensors into aluminum during casting is challenging due to the high process temperature as well as the high coefficients of thermal expansion (CTE) of the host material. Aluminum has a CTE of about 21 × 10^−6^ K^−1^, whereas silicon-based sensors have a CTE of 2.6 × 10^−6^ K^−1^. This inconsistency between the two materials results in high failure rates caused by thermally induced mechanical stress during the cooling of the cast component [2,3,4].

To measure mechanical load, Carlsson et al. embedded tungsten and titanium threads in aluminum and iron, which protrude out of the casting at both ends. One end of the thread is oscillated, while on the other, frequency and amplitude are logged. The initial results showed that it is possible to measure mechanical loads in a cast metal system with this approach [5,6]. Another measurement technique involving piezoelectric sensors was adopted by Rübner et al. and Klassen et al. [7,8]. The integration of thin film mechanical sensors in high pressure die casting (HPDC) was developed by Pille; under close examination, Pille proved that the sensing function was retained, though process-induced changes in sensor characteristics were observed [9]. Ibragimov et al. demonstrated the integration of a thermogenerator made of a borosilicate glass substrate via conventional microsystem technologies used in aluminum high pressure die cast processes [10].

In general, an embedded sensor must be considered a foreign body in the surrounding metal matrix if there is a mismatch in CTE, in the deviant modulus of elasticity, or in thermal conductivity [11,12]. Using aluminum as a substrate for sensors seems to be an appropriate solution for minimizing thermal and mechanically induced stress in the aluminum metal matrix; however, it introduces additional challenges.

Since aluminum is electrically conductive, the sensor needs electrical insulation from the substrate as well as from the cast aluminum. Common electrical insulations used by the semiconductor industry, such as silicon oxide or silicon nitride, are characterized by CTE values below 5 × 10^−6^ K^−1^ and brittle failure under tensile load, leading to the formation of cracks in the insulation layer at elevated temperatures. By using screen printed thick-film paste with a CTE adapted to that of aluminum, a reliable insulation can be produced that is capable of withstanding the harsh casting process in both gravity die casting and high pressure die casting [2,13]. This advantageous thick-film insulation was combined with a platinum thin-film strain gauge and was designed as a Wheatstone full bridge in this study. The combination of thick-film screen printing and thin-film micromachining has been used in previous studies [14], but using aluminum as a substrate and integrating the system obtained in an aluminum casting represents a novel approach in this context.

## 2. Materials and Methods

**Figure 1:**
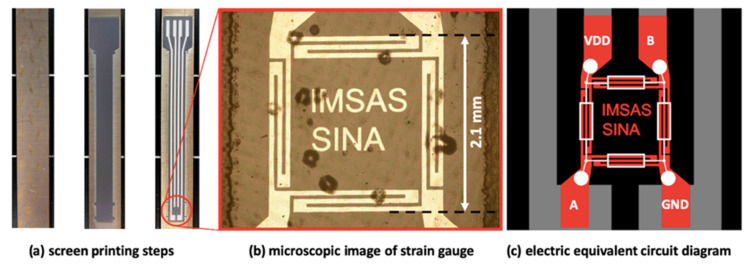
Design overview: (**a**) an aluminum sheet, an aluminum sheet with ground insulation, a sheet with screen printed insulation and a screen printed conductor; (**b**) a magnified cutting of the real sensor; (**c**) a circuit diagram of the full bridge strain gauge.

An aluminum sheet was used as a substrate; we ensured it was not scratched or dented above 50 µm for the thick-film insulation to perform properly. Screen-printable thick-film pastes produced by Heraeus (Hanau, Germany) were used for insulation and for a conductor [15]. The insulation to the substrate consisted of three layers, which were individually printed, dried, and fired in a furnace. The same ink was used for the top insulation. The insulation ink used was a ceramic ink with the serial number IP6080A [16]. The ink that acted as the conductor consisted of silver particles with the serial number C8829D [17]; it was used to print one layer, which was interrupted to integrate a thin-film full bridge strain gauge. The strain gauge consisted of a platinum thin-film, which was applied by physical vapor deposition and was structured via a lift-off process. An overview of the design can be seen in Figure 1. To separate the sensor from the cast aluminum, a top insulation was applied to the sensor. This top insulation was required to enclose the functional layer of the sensor and shield it from the surrounding cast material.

### 2.1. Screen Printing Process

**Figure 2:**
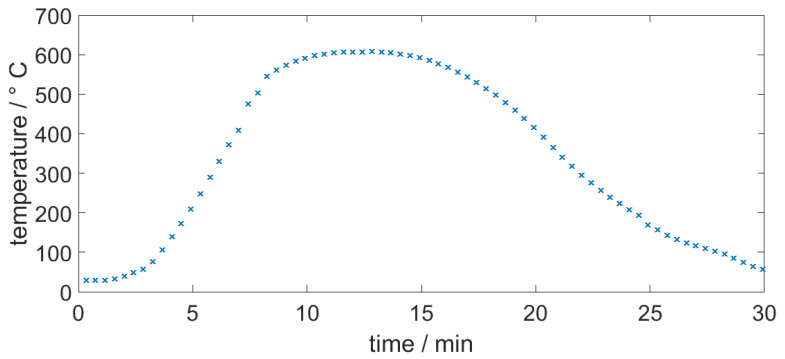
The measured temperature profile of the furnace for firing ground insulation.

Each thick-film layer was applied by screen printing using a disposable nylon mesh with a 45 µm opening and a thread diameter of 34 µm with 120 threads per inch. After printing, the ink was levelled for 10 min at room temperature and then dried for 15 min at 150 °C to remove the solvents. The dried layer was subsequently fired in a furnace at a temperature of approximately 600 °C for 10 min with a dwell time of 30 min. Each layer was printed, dried, and fired individually before the next layer was applied. The thick-film silver ink conductive paths were interrupted to leave room for insertion of the thin-film full bridge strain gauge; this will be described in Section 2.2. A screen-printed conductive silver ink was then used as a direct electrical connection. After the strain gauge was applied, the top insulation was deposited. The top insulation consisted of two screen printed layers that had been fired in a nitrogen atmosphere. The nitrogen atmosphere prevented oxidation of the thin-film strain gauge while it was exposed to the environment prior to full enclosure by the consolidated top insulation. The temperature profile of the furnace is depicted in Figure 2.

### 2.2. Sputtered Thin-Film Strain Gauge

**Figure 3:**
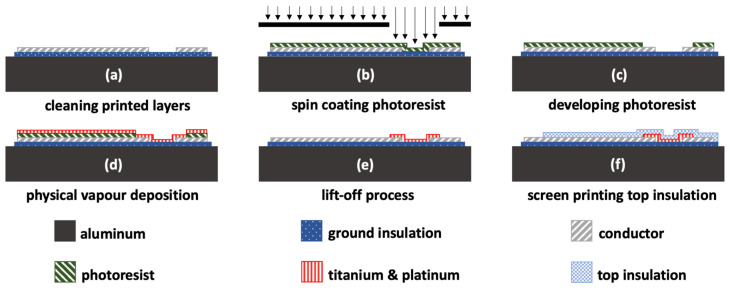
The schematic micro structuring process of the thin-film strain gauge on a thick-film surface with a subsequent screen printed top insulation step.

In this section, the steps for applying and micro-structuring the platinum strain gauge are described. The strain gauge element consisted of a 150 nm thick platinum metallization and a 10 nm thick adhesion layer of titanium. The full process can be seen in Figure 3.

The steps involved in the process depicted in Figure 3 included the following:

- Cleaning the screen-printed insulation and conductor with isopropanol from particles;
- Spin coating a 1.8 µm thick positive photoresist layer that had been heated on a hotplate at 100 °C for 30 s prior to exposure to UV light;
- Developing the positive photoresist; the thick-film surface that should be coated with thin-film metal is not covered with photoresist;
- Ensuring a physical vapor deposition (PVD) of the thin-film strain gauge consisting of a 10 nm thick adhesion layer of titanium and a 150 nm thick layer of platinum;
- Dissolving the photoresist as part of the lift-off process with acetone and cleaning with isopropanol; and
- Encapsulating the structured thin-film strain gauge with two additional screen-printed layers of the ink that was used for the ground insulation; which were fired under a nitrogen atmosphere, as described in Section 2.1.

### 2.3. Gravity Die Cast Embedding Process

**Figure 4:**
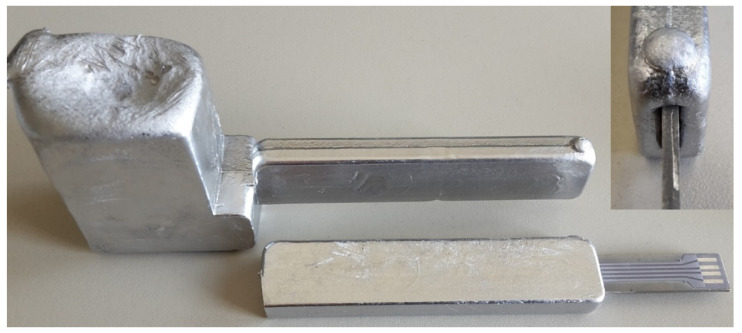
The filled cavity without the embedded sensor as well as the embedded sensor separated from the sprue (top right corner) in a detailed view of the protruding substrate.

Following this build-up process, the sensor was embedded in gravity die-casting using an AlSi_10_MnMg [18] alloy at a melt temperature of 720 °C. The die was preheated to 350 °C prior to casting to improve castability and to support the mold filling. In Figure 4, both the resulting cast body and the embedded sensor specimen are shown, with the former without the embedded sensor but with the sprue. The sensor substrate was 2 mm thick and 11.9 mm wide, and was positioned in the center of the casting. The specimen was 10 mm thick and 21 mm wide, and thus engulfed the sensor with 4 mm of aluminum.

## 3. Results

This section is divided in a characterization of the sensor sheet prior to the embedding process and a section providing data after the embedding process.

### 3.1. Sensor Sheet Characterization before Casting

**Figure 5:**
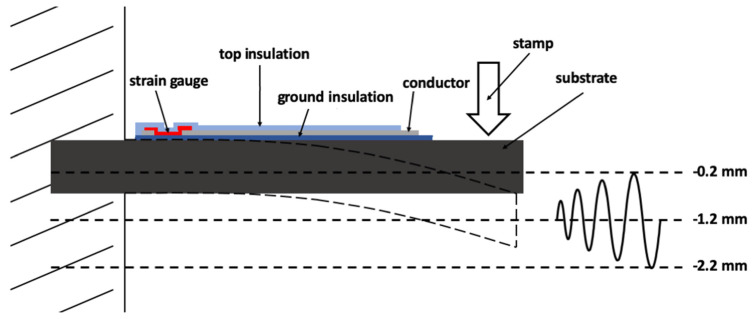
A schematic representation of the transverse beam test for sensor characterization prior to embedding.

The mechanical tests were performed as transverse beam tests prior to embedding and as a three-point bending test after the embedding process. The sensor was mounted in a horizontal position and a stamp deflected the beam in a specified manner. The test setup is displayed schematically in Figure 5.

When using a transverse beam test to observe the sensor signal, the stamp deflected the sensor to a center position at −1.2 mm with an amplitude of 1 mm. Thus, the sensor sheet was deflected by a minimum of −0.2 mm and a maximum of −2.2 mm, and an unloaded beam stood at the position of 0 mm.

**Figure 6:**
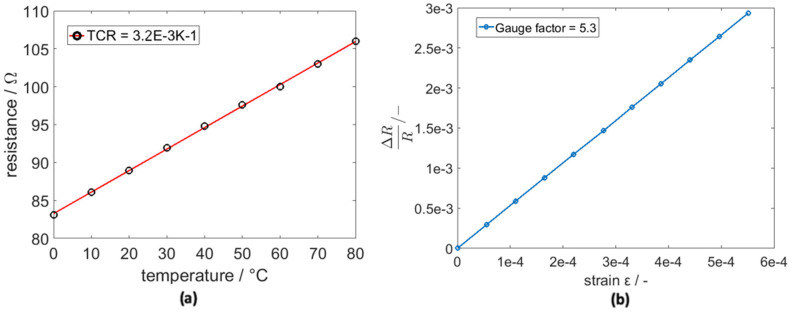
(**a**) The change in resistance with the temperature of the sensor; (**b**) the rate of resistance change due to the tensile strain of the sensor.

Using a climate chamber and measuring the resistance of the bridge, the sensor´s temperature coefficient of resistance (TCR) was calculated. This was executed with the slope of the linear fit and a resistance of 89 Ω at 20 °C. The calculated TCR was α_20__°C_ = 3.2 × 10^−3^ K^−1^, which was lower than in bulk material with α_20__°C_ = 3.8 × 10^−3^ K^−1^. Thin films have a higher resistivity than bulk material due to their smaller density and other effects, such as the collision of an electron with the surface. These additional effects on resistivity do not scale with higher temperature; hence, the TCR of a thin film will consistently be lower than that of bulk materials [19]. The temperature measurement graph in Figure 6 demonstrates our findings. With a COMSOL transverse beam FEM-Model, we calculated the elongation difference (strain) between −0.2 and −2.2 mm deflection. With a resistance difference measurement, the gauge factor (k) of the sensor was derived as 5.3. Literature regarding the gauge factor of platinum is not specific, but gauge factors can be found from k = 2 to k = 6, depending on the processing parameters. The plot is displayed in Figure 6.

**Figure 7:**
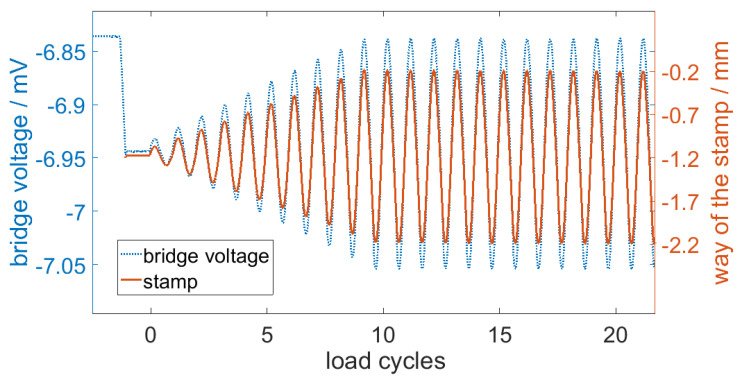
The measurement signal of the bridge voltage with the way of the stamp.

Initially, the stamp assumed the center position of 1.2 mm and faded into the maximum amplitude of 1 mm to bend the sensor. These measurements were recorded by applying 800 mV to VDD and measuring the bridge voltage between A and B, as shown in the design section in Figure 1c. In Figure 7, the first load cycles are presented. The diagram underlines that the measured bridge voltage accurately fit the deflection of the sensor.

**Figure 8:**
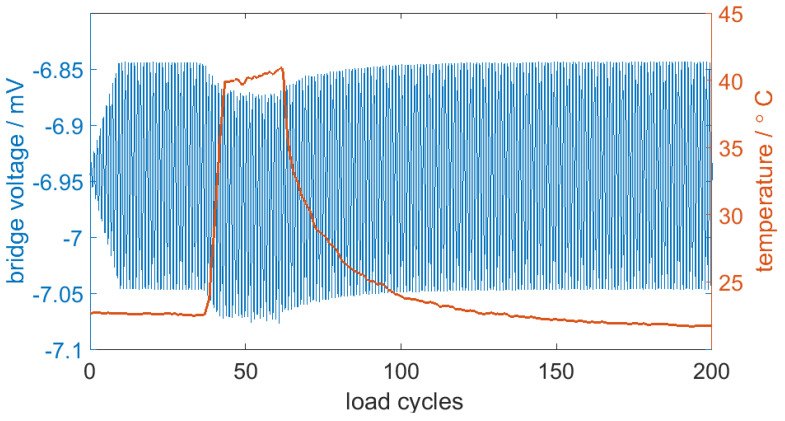
The measured bridge signal with the heat impulse; a 25 °C heat impulse led to a 10% bridge voltage change.

Figure 8 shows the results we obtained from subjecting the sensor and bridge to a heat impulse of 25 °C: the bridge signal changed by 0.025 mV in this 25 °C heat change. This can be translated into a repeatable value of 0.4% bridge voltage change per °C.

### 3.2. Results after the Embedding Process

**Figure 9:**
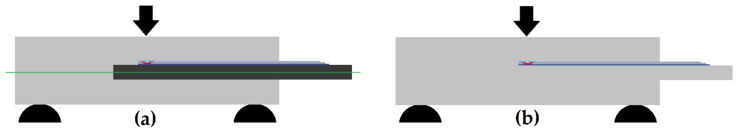
(**a**) A schematic cross view of the sensor with the neutral fiber and the substrate; (**b**) a schematic cross view of the sensor, only showing the foreign matter in the casting good.

Following the embedding process, the substrate was in the center of the specimen. In this position, the sensor was close to the neutral fiber, or plane, as shown in Figure 9. With a specimen thickness of 10 mm and an embedded substrate of 2 mm, the sensor was 1 mm out of the neutral fiber, which was sufficient to detect a strain signal under bending loads. We used a three-point bending setup to test the specimen mechanically.

**Figure 10:**
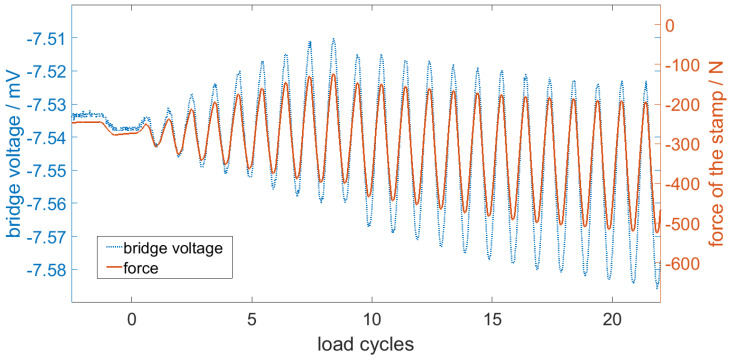
The measurement signal of the bridge and the force of the stamp in a three-point bending test.

The sensor resistance increased about 2.5% as result of the embedding process and thus the TCR changed slightly from α_20__°C_ = 3.2 × 10^−3^ K^−1^ to α_20__°C_ = 3.1 × 10^−3^ K^−1^. The TCR was determined in a climate chamber test by measuring the resistance, with longer holding periods at given temperature levels to consider the larger thermal capacity of the specimen. The data confirmed that the sensor can be used to measure the mechanical strain in the form of compression and elongation applied to the specimen. In Figure 10, the first load cycles from the three-point bending tests are shown in detail.

**Figure 11:**
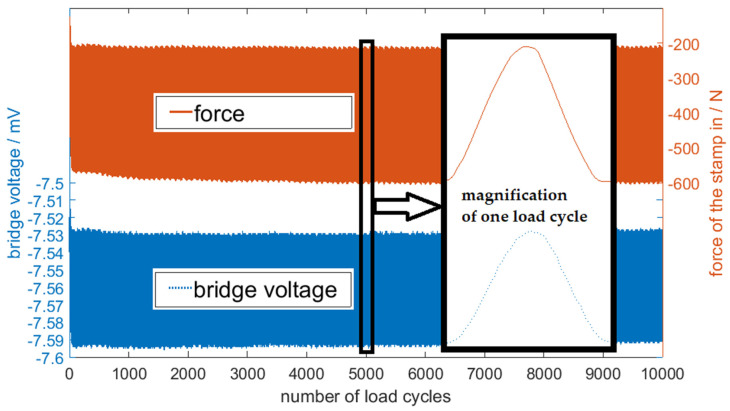
The measurement signal of 10,000 load cycles in a three-point bending test.

Within these first load cycles, the bridge voltage and the force of the stamp did not run parallel. After about 100 load cycles, the measurement signal was stable and synchronized. We performed over 35,000 load cycles in total, including compression as well as tensional load. Figure 11 shows a set of 10,000 load cycles.

## 4. Discussion

This paper presented the design, synthesis, and characterization of a piezoresistive strain gauge on an aluminum substrate for subsequent embedding in an aluminum casting process. The sensor was capable of measuring the mechanical loads acting on the casting. The initial load cycles after the embedding process showed a difference between the force of the stamp and the bridge voltage signal. Both the embedded sheet and the whole casting good showed that they might reduce the residual stress that built up during casting. After these initial load cycles, we were able to gain a stable measurement signal from the sensor, since the measured signal directly correlated to the force of the stamp and showed no drift for over 35,000 load cycles. The mechanical load of the casting good was measured as tensional as well as compressional load. Thus, the thin-film/thick-film technology combination that we used to realize an aluminum-based strain gauge for integration in aluminum castings seemed to be promising.

In future studies, the screen-printed thick-film pastes can be replaced by sufficient thin-film layers to further reduce foreign matter. The screen printed thick-film silver conductor can be replaced by an additional layer of PVD platinum with a sufficient thickness to achieve a low resistance and to supersede the thick-film ink as a conductor. Ground- and top-insulation may be substituted by a thin-film layer of aluminum oxide. With an aluminum CTE of about 21 × 10^−6^ K^−1^ and an aluminum oxide CTE of 10 × 10^−6^ K^−1^, the mismatch in CTE causes cracks due to thermally induced mechanical stress. Whereas the silicon substrate typically fails during cooling, an aluminum oxide layer will fail due to high tensile stresses induced by the expansion of the substrate during the inflow of the melt. A possible solution may be to use an aluminum oxide layer with intentional compressive residual stress to partly compensate high tensile stress acting on the ceramic insulation. A highly dense layer of aluminum oxide from a high-power impulse magnetron sputtering (HiPIMS) process deposited at an aluminum substrate temperature of 350 °C or higher could serve this purpose.

Instead of the embedded Wheatstone bridge, which has hard-wired temperature compensation, a sensor consisting of two meander-like geometries made from different materials, such as platinum and nickel with widely deviating TCRs, could be used to calculate strain and temperature.

## References

[1] Lehmhus D., Busse M., Bosse S., Lehmhus D., Lang W., Busse M. (2018). Structural Health Monitoring. Material-integrated intelligent Systems:Technology and Applications.

[2] Dumstorff G., Pille C., Tiedemann R., Busse M., Lang W. (2017). Smart aluminum components: Printed sensors for integration into aluminum during high-pressure casting. J. Manuf. Process..

[3] Kobliska J., Ostojic P., Cheng X., Zhang X., Choi H., Yang Y., Li X. Rapid fabrication of Smart Tooling with Embedded Sensors by Casting in Molds Made by Three Dimensional Printing. Proceedings of the 2005 International Solid Freeform Fabrication Symposium.

[4] Choi H., Datta A., Cheng X., Li X. (2006). Microfabrication and Characterization of Metal-Embedded Thin-Film Thermomechanical Microsensors for Applications in Hostile Manufacturing Environments. J. Microelectromech. Syst..

[5] Carlsson R., Elmquist L., Thore A., Ahrentrop F., Johansson C., Israelsson B. Connecting sensors inside smart castings. Proceedings of the 7th International Symposium on Aircraft Materials 2018.

[6] Carlsson R., Thore A., Elmquist L., Johansson C., Ahrentrop F., Schaller V., Johannisson P., Israelsson B., Törnvall M., Zander P. Sensors integrated inside metal castings verified to respond to force. Proceedings of the IX ECCOMAS Thematic Conference on Smart Structures and Materials 2019.

[7] Rübner M., Körner C., Singer R.F. (2008). Integration of Piezoceramic Modules into Die Castings—Procedure and Functionalities. Adv. Sci. Technol..

[8] Klassen A., Rübner M., Ilg J., Rupitsch S.J., Lerch R., Singer R.F., Körner C. (2012). Influence of the fabrication process on the functionality of piezoceramic patch transducers embedded in aluminum die castings. Smart Mater. Struct..

[9] Pille C. In-Process Embedding of Piezo Sensors and RFID Transponders into Cast Parts for Autonomous Manufacturing Logisitics. Proceedings of the Smart Systems Integration 2010.

[10] Ibragimov A., Pleteit H., Pille C., Lang W. (2012). A Thermoelectric Energy Harvester Directly Embedded Into Casted Aluminum. Electron Device Lett..

[11] Dumstorff G., Paul S., Lang W. (2014). Integration without Disruption: The Basic Challenge of Sensor Integration. IEEE Sens. J..

[12] Bosse S., Lehmhus D., Bosse S., Lehmhus D., Lang W., Busse M. (2018). On Concepts and Challenges of Realizing Material-Integrated Intelligent Systems. Material-Integrated Intelligent Systems—Technology and Applications.

[13] Tiedemann R., Fischer M., Busse M., Lang W. (2018). Integrating sensors in castings made of aluminum—New approaches for direct sensor integration in gravity die casting. Procedia Manuf..

[14] Vincenzi D., Butturi M.A., Stefancich M., Malagù C., Guidi V., Carotta M.C., Martinelli G., Guarnieri V., Brida S., Margesin B. (2001). Low-power thick-film gas sensor obtained by a combination of screen printing and micromachining techniques. Thin Solid Film..

[15] Heraeus Celcion—Materials System for LED Circuits. https://www.heraeus.com/media/media/het/doc_het/products_and_solutions_het_documents/thick_film/Brochure_Heraeus_Celcion_-_Materials_System_for_LED_Circuits.pdf.

[16] Dielectrics_IP6080A. https://www.heraeus.com/media/media/het/doc_het/products_and_solutions_het_documents/thick_film/thick_film_data_sheets/hybrid_electronics/alternative_substrates/Dielectrics_IP6080A.pdf.

[17] Conductors_C8829D. https://www.heraeus.com/media/media/het/doc_het/products_and_solutions_het_documents/thick_film/thick_film_data_sheets/hybrid_electronics/alternative_substrates/Conductors_C8829D.pdf.

[18] Silafont-36. https://rheinfelden-alloys.eu/wp-content/uploads/2016/01/05-Sf-36-Merkmale-RHEINFELDEN-ALLOYS-2016.pdf.

[19] Lang W. (2019). Sensors and Measurement Systems.

